# In Vitro Models Used in the Formation of Root Caries Lesions—A Review of the Literature

**DOI:** 10.3390/dj11120269

**Published:** 2023-11-26

**Authors:** Zaid Dohan, Lara T. Friedlander, Paul R. Cooper, Kai-Chun Li, Jithendra T. Ratnayake, May L. Mei

**Affiliations:** Sir John Walsh Research Institute, Faculty of Dentistry, University of Otago, Dunedin 9016, New Zealand; zaid.dohan@otago.ac.nz (Z.D.); lara.friedlander@otago.ac.nz (L.T.F.); kc.li@otago.ac.nz (K.-C.L.); jithendra.ratnayake@otago.ac.nz (J.T.R.)

**Keywords:** in vitro, root caries lesion, dentine, cementum, demineralisation, remineralisation

## Abstract

The management of root caries remains a challenge for clinicians due to its unique anatomical location and structure. There is increasing interest in utilising artificial root caries lesions to develop new strategies for remineralisation. An ideal protocol has not yet been agreed upon. The aim of this review is to provide a structured overview of previously reported in vitro root caries models. The literature was screened and mined for information mainly on substrate selection, model systems utilised, and variables used in the models. Human roots (60%) were the most frequently used substrates, followed by bovine roots (40%). Chemical models (69%) were the most frequently utilised model systems, followed by microbiological models (27%), to form root caries lesions. Acetate buffer solution (80%), pH 5.0 or above (40%), and a demineralisation time of five days (25%) were the common variables used in the chemical systems, while mono-species biofilm was most frequently used (73%) in microbiological models and *Streptococcus mutans* was the most common bacterial strain utilised in these models (80%). This review highlights the variability amongst the experimental approaches, discusses the advantages and limitations of these approaches, and emphasises that standardisation of experimental conditions along with sustained research will benefit root caries research.

## 1. Introduction

Dental root caries has been defined as caries that begins on the cemental root surface in the oral environment [1]. Root caries lesions are often diagnosed in patients of advanced age. This is due to hyposalivation and root exposure caused by gingival recession as a result of ageing [2,3]. A high prevalence of root caries has been reported globally as the number and proportion of people over the age of 65 has increased [4], with approximately 33% of the elderly population reported as being affected by root caries [5,6].

Increased prevalence of root caries in the population has led to increased studies investigating root caries and its potential treatments. In the absence of chronic periodontal disease, dentine root surfaces are usually covered by a relatively thin layer of cementum, which is softer and more porous than enamel. In addition, the irregular anatomical topography of the cementum enamel junction (CEJ) contour provides a site for microbial retention and development of biofilm [7]. Root caries lesions are softened, discoloured, may or may not be cavitated, and have a tendency to spread laterally, making them shallow yet extensive [8]. Cavitation is not a sole indicator of lesion activity, as both cavitated and non-cavitated lesions can be either active or arrested in root surfaces [9]. Lesions can also spread subgingivally and proximally, making access, isolation, and restoration difficult, and thus prevention or reversal of these lesions in the initial stages is particularly important [8]. Most caries preventive agents have been initially studied using in vitro mechanistic studies, i.e., devices and drugs [10,11]. In vitro models allow relatively rapid development of new treatments, since the experimental conditions can be well controlled, less complex, and standardised, and the experimental durations are much shorter than in clinical trials [11,12,13].

Artificial caries lesions mimic clinically incipient root caries to which treatment will be applied. A successfully formed and reproducible in vitro lesion is fundamental to enable comparisons of treatments and assessment of outcomes [11]. There are several factors which can be manipulated when inducing these lesions, including the different types of chemicals, acids, or microbes used; the level of acidity; and the duration of exposure. Consequently, there are many variables between studies, yet no consensus has been reached to identify an optimal and standardised methodology. The current review is the first of its kind and aims to assess and provide an overview of the current literature on the different in vitro models used to form artificial root caries lesions.

## 2. Materials and Methods

### 2.1. Literature Search Strategy

A search of the literature was undertaken in PubMed to identify English-language publications from 2002 to 2022. The keywords used included: (in vitro) AND (root caries).

### 2.2. Study Inclusion and Exclusion Criteria

#### 2.2.1. Inclusion Criteria

(1) In vitro studies.
(2) Studies on primary root caries.
(3) Studies which utilised root surfaces or root dentine.
(4) Studies which used chemical or microbiological models to induce or attempt to induce root caries formation.

#### 2.2.2. Exclusion Criteria

(1) Articles not written in English.
(2) Studies which used coronal dentine.
(3) Studies on secondary root caries following placement of restorations.
(4) Studies inducing erosion rather than caries or demineralisation.
(5) Full article texts unavailable.
(6) Case reports, conference papers, book chapters, patents, letters to the editor, systematic reviews, meta-analyses, and literature review papers.

## 3. Results

**Figure 1:**
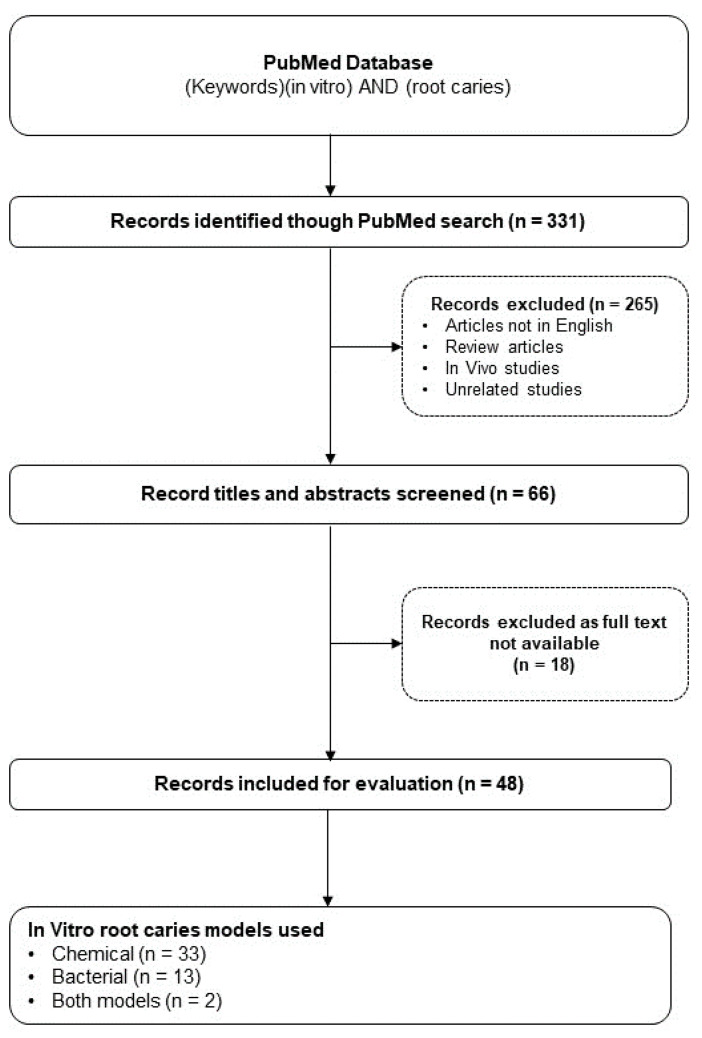
PRISMA flow chart using search terms ‘root caries’ and ‘in vitro’ from 2002 to November 2022 on the PubMed database.

**Figure 2:**
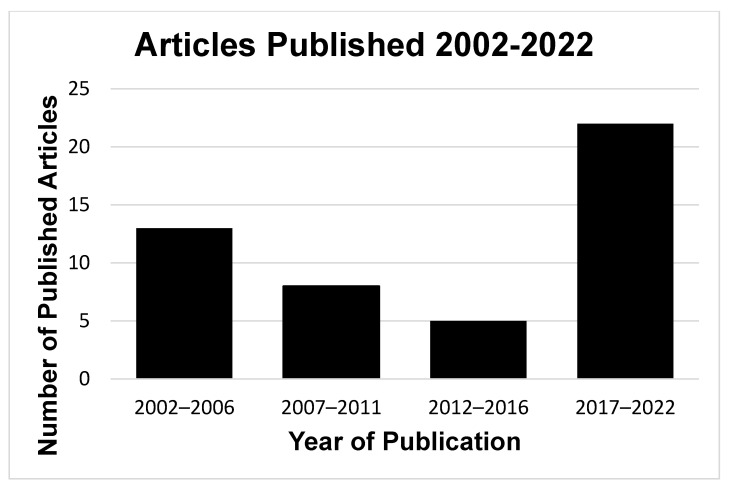
Frequency distribution of original research articles which studied induced root caries in vitro identified from PubMed using the search terms described in the text. These are grouped in 5-year intervals over the previous 20 years, from 2002 to 2022.

A total of 331 potentially eligible articles published over the 20 years up to November 2022 were identified using the search terms described. After screening titles and abstracts, 66 articles remained for further analysis. Full-text reading was undertaken, and 48 articles were finalised for inclusion in this review (Figure 1). The number of articles published initially declined from 2002 to 2016 when they were grouped in five-year intervals; however, there appears to have been increased research activity in this area more recently, from 2017 to 2022 (Figure 2).

**Figure 3:**
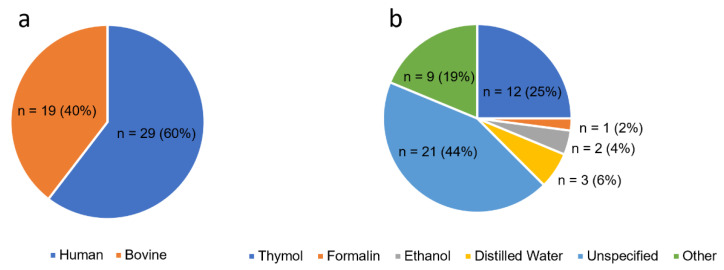
Pie chart of tooth substrates used in the studies reported in this review (**a**). Pie chart showing the frequency of use of storage media or methods of tooth storage following extractions reported (**b**).

Either bovine or human tooth roots were used as substrates for the generation of root caries lesions, with the majority of studies using human tissue (60%) (Figure 3a). Among the studies, 19 specified whether the cementum remained intact or was removed from the root surface. Storage of teeth prior to use was mostly not reported in 21 studies (Figure 3b). One quarter of studies specified that specimens were stored was in a thymol–water solution in a concentration range of 0.08–1.0%. One study utilised a thymol–alcohol mix, while another study reported the use of a saline-containing thymol solution. Other solutions used for storage included formalin, sodium azide, 5% sodium hypochlorite, and ethanol. One study refrigerated specimens, whilst two studies froze specimens at −20 °C prior to analysis. Two studies described the immediate use of specimens following collection without storage, and one other study stored specimens in an airtight humidified container prior to use. 

**Figure 4:**
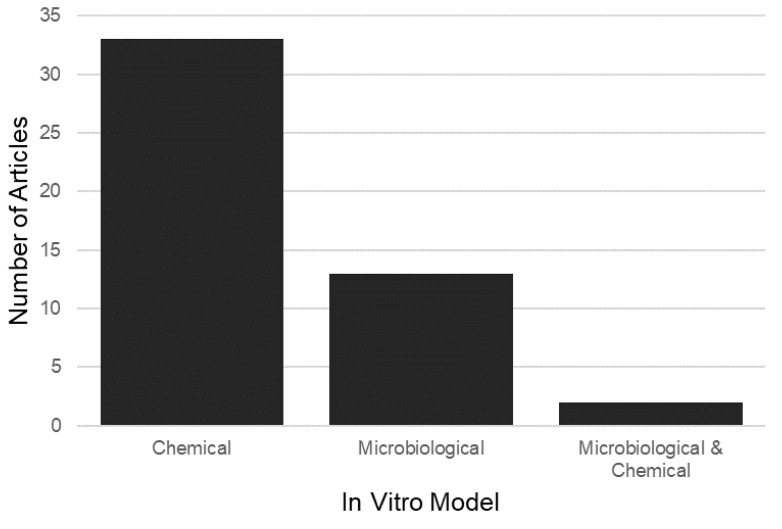
Model type and frequency of use in articles included for review with the search terms described.

Chemical, microbiological, or a combination of both methods were used to induce artificial root caries (Figure 4). Chemical exposures were mostly utilised to create root caries lesions (69%). Of the studies, 33 exclusively used a chemical model system, whilst 2 studies used a combined chemical and microbiological model system. A microbiological model was exclusively used in 13 studies (27%).

The chemical model parameters used in all the studies to induce root lesions are provided in a–c in Table 1. An acid solution was used in 29 studies, with 28 using acetic acid and 2 using lactic acid solutions. Acidic gel systems were also utilised, though to a lesser extent, with a lactic acid gel being used in five studies. One study utilised acetic acid, whilst one used a phosphoric acid gel. The pH values of the acids (gels or solutions) ranged from 4.0 to 5.46, with 40% of studies reporting the use of pH 5.0 or higher to induce root lesions. The exposure time taken to induce lesions ranged from 15 s to 35 days, and this was dependent on the different acids used.

The microbiological model system parameters used to create root caries lesions are described in a and b in Table 2. Among the microbiological models used, a mono-species bacterial culture system was used in 11 studies. *Streptococcus mutans* was the most common bacterial strain applied (12 studies), followed by *Lactobacillus rhamnoses*, which was applied in 4 studies. Dual-species, tri-species, and multi-species cultures were used to lesser degrees to induce lesions. A total of seven different bacterial strains were used.

**Figure 5:**
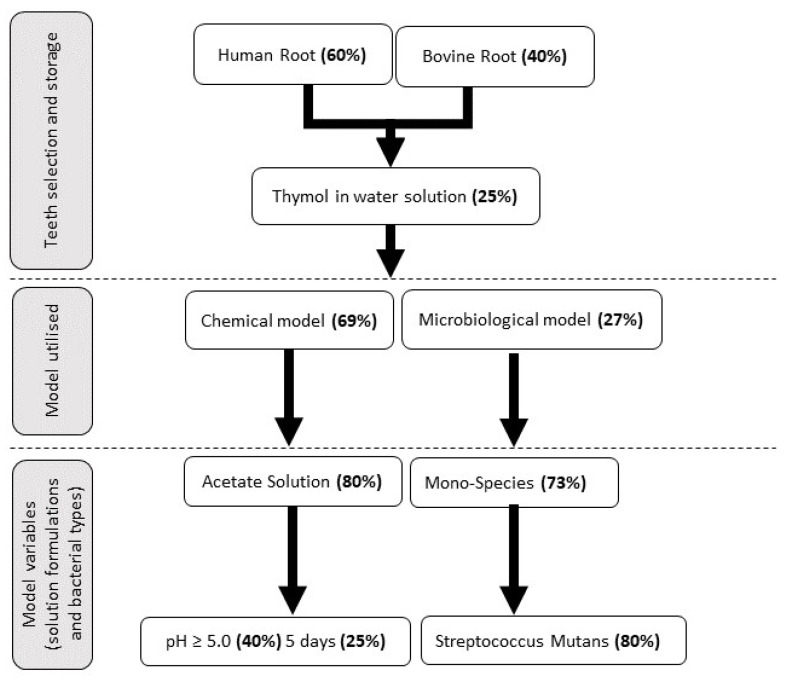
Flow chart showing frequencies of research methodologies used in in vitro studies for root caries model systems. The percentage values shown in the flow chart indicate the most frequently used procedures.

To further summarise the findings from all the different model systems reported, a flow chart has been generated providing a guide for the in vitro models developed and used by researchers working in the field (Figure 5).

## 4. Discussion

This review has assessed and provided an overview of different in vitro models used and reported in the literature for the formation of dental root caries. Notably, a range of methods and strategies are employed based on the capabilities and resources present in research laboratories around the world, which will be discussed.

A total of 60% of the reviewed studies used human roots as the substrates for root caries research [4,14,15,16,17,18,19,20,21,22,23,24,25,26,27,28,29,30,31,32,33,34,35,36,37,38,39,40,41]. The benefits of using human teeth are that they provide findings more translatable to clinical practice, since the substrate used is the human root, which reflects the clinical situation. Bovine roots were the second common substrates used in the reviewed articles [2,10,42,43,44,45,46,47,48,49,50,51,52,53,54,55,56,57,58]. Bovine dentine has been a valuable substrate used in research methodologies for the prevention or treatment of dentinal carious lesions [58]. There are, however, some differences reported between bovine and human dentine, as highlighted in a review by Yassen et al., in 2011, where the authors found inconsistencies in the data comparing bovine dentine and human dentine in previous studies, in particular, morphological, chemical composition, and physical property differences between the two substrates [59]. A more recent study compared the effects of lesion baseline severity, mineral distribution, and substrate on the remineralisation and progression of in vitro caries lesions created in root dentine [60]. Some differences were found between human and bovine dentine and their relative responsiveness to de- and remineralisation, though the effects of lesion baseline mineral distribution and severity were similar between both substrates [60]. Indeed, it is reported that the biological differences between human and bovine dentine are relatively minor, and therefore bovine dentine provides a representative substrate in mechanistic root caries studies. In addition, it is worth noting that bovine teeth usually do not need separate ethical approval if the animals are part of a regulated food-chain system, which could make tooth collection effective. Bovine incisors have larger root surfaces compared to human root dentine, which also makes specimen preparation less technique-sensitive.

Inadequate or inappropriate storage of teeth can affect the microhardness and quality of dentine, as well as the cleanliness of specimens [61]. Antimicrobial solutions are used for disinfection and as storage media for teeth prior to testing [61]. Ideally, an antimicrobial solution should eliminate all bacteria, viruses, fungi, and spores from the teeth and prevent the growth of pathogenic colonies without affecting the quality and structure of the enamel or dentine when stored over time [61]. This review demonstrated that there are numerous types of storage approaches that are utilised and reported in the literature, including the use of solutions of formalin, sodium azide, sodium hypochlorite, ethanol, and thymol. Notably, there is a concern that antimicrobial solutions can have adverse effects on the mineral content and compositional structure of dentine [62]. The most common method used to prevent bacterial contamination in extracted teeth was to store them in a thymol solution [4,23,30,33,34,35,36,37,38,39,54,56]. Thymol causes the perforation of cell membranes, resulting in bacterial cell death [63]. Immersion in a 0.1% thymol solution has been shown to completely eradicate *Streptococcus mutans* from artificial sub-surface root caries lesions, though its use cannot ensure the eradication of all pathogens, including viruses, spores, and even prions, which may be present in teeth [26]. Polishing of tooth specimens to expose fresh surfaces prior to treatment could potentially remove contamination and provide standardised surfaces for caries lesion generation.

Root dentine is relatively easily accessed by cariogenic stimuli due to the comparative thinness of the overlying cementum layer, which, when removed, subsequently exposes the dentine to risk of mineral loss [64]. The thickness of cementum is only 20 to 50 µm around the CEJ, where root caries usually initiates [65]. Consequently, the cementum layer has been postulated to provide initial caries resistance for the root surface [15]. Less than half of the studies included in this review mentioned or specified if cementum was intact or removed prior to lesion induction [4,15,17,18,19,21,22,23,24,25,26,28,31,39,43,45,46,47,51]. Smith et al. demonstrated that cementum removal resulted in the formation of larger, deeper lesions and proposed that this was due to a reduction in the demineralisation rate and the permeability of cementum compared with dentine [23]. However, cementum is typically eroded by the time caries develops in clinical situations, and the removal of cementum from tooth roots used in in vitro studies would enable the standardisation of dentine samples and reduce variability [39]. Notably, clinically, tooth roots frequently have a thin layer of superficially hypermineralised cementum, which can be removed by intensive root planing or toothbrushing [46,55].

Chemical in vitro models have been developed to provide relatively simple and reproducible models for caries. Despite these systems not using bacteria and degradative enzymes, they can create lesions histologically similar to those found in natural root caries, with comparable depths [29].

Lactate solutions have been used as demineralising agents to simulate the effect of cariogenic bacteria, such as lactobacilli, which produce lactic acid [66,67]. While lactate treatment is highly effective at removing minerals from the collagenous dentine matrix and produces lesions closer in composition to natural caries [66], this review found that acetate buffers were the most frequently utilised demineralising solutions [4,14,16,17,18,22,23,26,28,29,31,35,37,38,39,42,44,45,46,47,48,49,50,51,52,54,55,57]. This finding may be because acetate solutions, at the same concentration and pH, are able to demineralise dentine at a more rapid rate than lactate [66].

Gel systems were identified as the second most-common chemical system following acidic solutions [15,20,21,23,27,41,43]. It is understood that gels restrict the diffusion of acid into a lesion and consequently result in a smaller lesion compared with a free-flowing acid solution [23]. Whilst acid solutions have been shown to produce relatively large, deep lesions with well-mineralised, thick surface layers, gel systems have been shown to produce lesions that are shallow, smaller in size, and with less well-mineralised surface layers [23]. Lesions also form more rapidly in acid solutions compared with acid gel systems, and these were more reliably produced when cementum was removed [23].

The pH of the acids used ranged from pH 4.0 to 5.46, with the majority of studies reporting the use of acids at pH 5.0 or above [14,15,20,31,37,42,43,45,47,48,50,51,52,54] to induce root lesions. This value is different from the common value of pH 4.4 used in enamel lesion formation. The reason behind this may be that the critical pH of root dentine ranges between 6.2 and 6.4, which is higher than the critical pH of enamel of 5.5 [68].

Apart from the acid component, the chemical solutions applied should also contain calcium and phosphate ions. This composition enables the mimicking of the process of caries in saliva. In the in vivo situation, caries occurs in the presence of calcium and phosphate ions, rather than simple demineralisation by acidic challenge [69]. One study in this review used phosphoric acid alone for 15 s to demineralise and mimic dentinal root caries [41]; however, this simple application of a strong acid would be expected to etch dentine rather than develop root caries [70].

Despite their relative simplicity, chemical models alone are not capable of protein denaturation, though microbiological models have been shown to have proteolytic activity in dentine [71]. Consequently, microbiological models have been created which utilise bacteria cultured on root dentine and are known to be important in the caries process in vivo. Cariogenic mono-species biofilms were found to be the most common microbiological models in this review [10,19,21,24,25,30,32,33,34,50,56]. Bacteria, such as *Streptococcus mutans (S. mutans*), Lactobacilli, and Actinomyces species, that are acidogenic and aciduric are inoculated for growth on tooth surfaces and are important in the aetiology of root caries lesions [72,73]. *S. mutans* exhibits collagenolytic activity and can invade the collagen-rich dentinal tubules [71,74]. Mello et al. created artificial root lesions by incubating tooth roots in *S. mutans* biofilm for 15 days and found that the root lesions formed were similar macroscopically and microscopically to in vivo lesions, as they displayed cementum loss and alterations of the underlying dentine, with the lesions located close to the CEJ [24]. Clinically, lactobacilli are also highly prevalent in root caries lesions [75]. Lactobacillus species were the second most-applied species reported in this review [10,19,25,36,50,58]. Geraldo-Martins et al. have also demonstrated that microbiological methods created dentine lesions that were very similar to caries lesions in both clinical and microscopic aspects [32].

Other studies used multi-species biofilms [2,36,53]. It is thought that this model type replicates the biodiversity of root caries as closely as possible [76,77]. However, oral biofilms in reality are more complex and are composed of hundreds of different bacterial species which may be difficult to grow or cultivate in a laboratory setting [73,76,78]. Consequently, it is understood that the demineralisation dynamics produced by natural biofilms differ from those of biofilms in vitro; therefore, exact modelling of cariogenic biofilms in vitro which represent the real situation is almost impossible, and therefore they have limited generalisability [19]. Microcosm biofilms are able to be grown directly from saliva or plaque and are reportedly much more diverse; however, they can be challenging to characterise, culture, and standardise [79]. The majority of studies identified in this review which utilised a microbiological model favoured mono-species selection, and only very few studies used plaque to induce root caries lesions in vitro. This is likely due to microbiological models being more complex, technique-sensitive, and time-consuming to undertake.

Lack of consistency and reproducibility can be a limitation of microbiological models when compared with chemical models. Microbiological models cannot easily be regulated by accurate control of acid, pH values, or mineral contents [12]. The parameter that can be controlled is the initial concentration of the microorganisms applied. Slight variations in the initial concentration may result in a significant difference later in the culture, since bacteria grow exponentially. Furthermore, when multi-species cultures are employed, the interaction between different species is also unpredictable. However, for studies investigating the effect of antimicrobial intervention, it is realistic to incorporate biological factors into the model systems to best mimic the dynamics of caries lesion development [80].

It has been demonstrated that non-cavitated root caries lesions are more likely to arrest compared with cavitated lesions [81,82]; therefore, it is crucial that studies specify whether the in vitro root caries lesions produced are cavitated or non-cavitated. It is evident that conducting more thorough analyses to determine the most suitable combinations of methodologies and approaches will lead to improved standardisation of processes. Moreover, it is unfeasible to cover all research areas related to this topic, and the exploration of other variables, for example, levels of calcium and phosphates in the chemical solutions, remains meaningful. Nevertheless, the information presented suggests that improved standardisation of experimental methods will ultimately result in substantial advancements and benefits for the field of caries research.

## 5. Conclusions

- This review provides a holistic overview which may facilitate and direct future studies that aim to induce in vitro root caries lesions.
- It is apparent that the methodology used for inducing these experimental lesions has evolved over the past two decades, although there remains no consensus regarding an ideal protocol.
- We have generated a guide to induce root caries lesions in in vitro model systems in this review.
- It is acknowledged, however, that alternative techniques and approaches may be employed based on the expertise and available resources in individual research laboratories.

## Figures and Tables

**Table 1:** (**a**) Frequencies of differing chemical model acid types used to induce root caries in articles in this review. Frequencies can be mutually inclusive, as more than one acid type was used in some studies. Percentages are rounded to the nearest whole number. (**b**) Frequencies of differing chemical model pH acid values used in articles in this review to induce root caries. Frequencies can be mutually inclusive, as more than one pH was used in some studies. Percentages are rounded to the nearest whole number. (**c**) Frequencies of time durations taken (days) to induce root caries in articles in this review. Frequencies can be mutually inclusive, as more than one duration was used in some studies. Percentages are rounded to the nearest whole number.

(a)	
Acid Type	Number of Articles n = 35 (Percentage)
**Solution**	29 (83%)
Acetic Acid	28 (80%)
Lactic Acid	2 (6%)
**Gel**	7 (20%)
Acetic Acid	1 (3%)
Lactic Acid	5 (14%)
Phosphoric Acid	1 (3%)
**(b)**	
**pH of Acids**	**Number of Articles n = 35 (Percentage)**
≤4.4	3 (8%)
4.5	10 (26%)
4.6	5 (13%)
4.7	2 (5%)
4.8	2 (5%)
4.9	2 (5%)
≥5.0	14 (37%)
**(c)**	
**Days to Induce Root Caries**	**Number of Articles n = 35 (Percentage)**
≤1	4 (8%)
2	4 (8%)
3	7 (13%)
4	8 (15%)
5	9 (17%)
6	1 (2%)
7	8 (15%)
14	4 (8%)
21	5 (9%)
≥28	3 (6%)

**Table 2:** (**a**) Frequencies of the types of microbiological biofilms used in this review. Frequencies can be mutually inclusive, as more than one biofilm was used in some studies. Percentages are rounded to the nearest whole number. (**b**) Frequencies of bacterial strains used in articles in this review. Frequencies can be mutually inclusive, as more than one strain was used in some studies. Percentages are rounded to the nearest whole number.

(a)	
Microbiological Biofilm Used	Number of Articles n = 15 (Percentage)
Mono-Species	11 (73%)
Co-Culture	2 (13%)
Tri-Species	3 (20%)
Multi-Species	3 (20%)
**(b)**	
**Bacterial Strain Used in Biofilm**	**Number of Articles n = 15 (Percentage)**
*Streptococcus mutans*	12 (80%)
*Streptococcus sobrinus*	1 (6%)
*Lactobacillus rhamnosus*	4 (26%)
*Lactobacillus acidophilus*	3 (20%)
*Actinomyces israeli*	2 (13%)
*Actinomyces naeslundi*	2 (13%)
*Actinomyces viscosus*	1 (6%)

## Data Availability

Not applicable.
